# Product-Centered Language Style in Chinese Women Online Reviews

**DOI:** 10.3389/fpsyg.2022.839064

**Published:** 2022-05-30

**Authors:** Xian Wang, Congjun Mu, Huixian Li, Alison Noble, Qingyi Wang

**Affiliations:** ^1^College of Foreign Languages, Shanghai Maritime University, Shanghai, China; ^2^Marine Engineering Department, Antwerp Maritime Academy, Antwerp, Belgium; ^3^ByteDance Ltd., Beijing, China

**Keywords:** language style, LSM, online reviews, women consumers, product content

## Abstract

The relationship of language style and online review has drawn increasing academic attention recently as it can provide customers with a guide to make the purchase. Extant research attaches importance to the language style that is presented in the use of function words, instead of product-related content words. This study aimed to examine the language style generated by customers’ comments relating to the product based on content words, that is, product-centered language style (PCLS). We built a corpus of Chinese women clothes online reviews to explore the general picture and distinct features of PCLS and the distinct feature of PCLS. A content-word-centered Linguistic Inquiry and Word Count (LIWC) in terms of product performance is established. PCLS is calculated based on the language style matching (LSM) algorithm. Our results show that the PCLS in women clothes online review is featured by diverse and polarized language styles among three groups of women clothes buyers, and the prioritized arrangement of words of importance contributes to the PCLS. The findings benefit the women clothes industry in which it can help companies quickly find the distinctive and the transition of PCLS and offer an approach for companies to indiscriminately look into the significance of the product category from the linguistic perspective, which can help with product sale strategy and product design.

## Introduction

Everyday, thousands of women consumers buy clothes based on previous online reviews (Carroll, 2008), and compared with men, their intention to purchase will increase if they receive a positive response (Garbarino and Strahilevitz, 2004). The review valence, review content, extremity and length, and language style are useful information that helps post purchaser make the decision (Forman et al., 2008; Mudambi and Schuff, 2010; Pan and Zhang, 2011). The language style of online product review has been identified as a constructive tool in management prediction, such as the third party’s evaluation of participants (Romero et al., 2015), the attenuated effect of low expertise level comments (Wu et al., 2017), and the way of emotional expressions (Liu et al., 2019). However, these business applications are achieved based on the analysis of emotions inherent in the language style, but none focuses on the specific way of expression when a certain group of customers refers to the product quality. In this study, we attempted to explore the features and influences of language style relating to product quality when Chinese women clothes consumers comment online on Taobao.

The language style of online reviews has been extensively researched in recent two decades either as an information signal (Romero et al., 2015; Liu et al., 2019) or as a genre of discourse (Teso et al., 2018; Cenni and Goethals, 2021). Language style may be a tool to measure the perceived result of consumers against previous consumers (Hong et al., 2017). While Guo et al. (2020) made effort to analyze the cognitive impact based on the non-emotional content, the majority of literature relies solely on the emotional language features to describe cognitive responses to the language style of online reviews among different information receivers (Antioco and Coussement, 2018; Liu et al., 2019, Guo et al., 2020; and etc.). In addition, some scholars state that the language style of online reviews is independent of the content of reviews and put emphasis on the tokenization of emotional linguistic features, that is, the emotion-centered language style (ECLS) (Swaab et al., 2011; Ludwig et al., 2013). In the field of discourse analysis, language style is studied with a focus on the function of an acknowledged discourse instead of the internal features. For example, Ren (2018) argued that there was a mitigated language style of online reviews, and the strategy for mitigation devices is related to the positive/negative review valence. Tseng and Zhang (2020), through the example of Chinese health communication, indirectly pointed out that language style in health online reviews has its own framework.

Despite these thorough studies on the language style of online reviews (OPCs) in business research and discourse analysis, factors identified to affect the language style regarding the product are mixed in the literature. Unclassified factors such as shopping experience, price, emotions, and non-emotions (Ma and Hu, 2012) proved to contribute to language style and product-centered language style (PCLS). Guo et al. (2020) scaled down the factors by separating non-emotional from emotional content but listed a mixture of non-emotion content that contains review valence, gender, credibility, and the scientific processing system of mankind. Product attributes such as length, rating valence, and affective content directly influence the review expressions (Forman et al., 2008), which is a mixed classification as well. From these efforts, it is clear that academia intends to detail the factors inherent in the language style of online reviews that synchronize that specific way of expression, that is, the product-centered language style. However, it is not satisfactory yet as to the question: to what extent are consumers influenced by previous comments purely relating to the product? We presumed this is caused by the following: (1) it is difficult to define the product attributes, as it is related to various aspects of the product, including the performance, material, and design. Recent academic studies relating to language style in the online review are controlled by function words reflecting the emotions (Fan and Chen, 2017; Liu et al., 2018; Guo et al., 2020). (2) Compared with the description of language style from function words, which is limited in a category and in quantity, it is quite challenging to describe the product from the content words. According to Tausczik and Pennebaker (2010), content words include nouns, regular verbs, adjectives, and adverbs. (3) There are no product-centered word categories available for the comparison of language style. To make up the imbalance, this study establishes a product-centered content-word-based Linguistic Inquiry and Word Count (LIWC), a dictionary based on categories, and explores the features of the language style of women clothes, purely on product aspects (PCLS), drawing on the corpus of Chinese women clothes online reviews. In particular, we addressed the following questions:

1. Is PCLS distinguishable among women clothes buyers?
2. What linguistic features make up the PCLS variations in women online reviews?

In what follows, we first reviewed the theory about the relationship of online review writers and post readers, the studies on PCLS. In the “Methods” section, we provided an overview of the approach called language style matching (LSM) and the new LIWC based on PCLS. In the “Results” section, we demonstrated the data and relevant explanations. In the “Discussion” section, we expound on the general features of PCLS of women reviews. Finally, we concluded that the PCLS is important to monitor and diagnose the value structure of companies’ products.

## Literature Review

### Language Expectancy Theory

The language used by review writers needs to consider the context of the relationship between the speaker and the audience (Pennebaker et al., 2003), as prior utterance affects the cognition of the recipients and may result in the synchronicity of language style, which is the linguistic scenario called the language expectancy theory (LET). LET posits that people develop appropriate communication styles in certain scenarios whereby they encode the language style of prior utterance, if affected, into their own communication styles (Burgoon and Miller, 1985).

The current academia in this regard focuses on comparing the language style matching between the speaker and the recipients in the context of different LET-related situations. Purposefully or unintentionally changing expectations will enhance or mitigate the effectiveness of the message whereby a given message will be incrementally enhanced, sharpening the recipients’ acceptance and their way of expression (Lee and Yu, 2020). Sentence structure or word choice positively or negatively influences the expectations of recipients (Averbeck and Miller, 2014). Linguistic expectations are compared to find the proper expression for crowdfunding (Parhankangas and Renko, 2017; Koh et al., 2020). In a natural disaster, the resharing of the information on Twitter plays an incremental role in self-strengthening and self-awakening (Lee and Yu, 2020). The stylistic choices made by an individual customer are intended to provoke a particular response from other customers (Semino and Culpeper, 2011). Furthermore, words are the key aspect to show the effect of language expectations, which results in the research on semantic features. For example, language density used by patients is compared to show that higher language density means the impact of certain topics on customers is more effective (Burgoon and Miller, 1985; Burgoon et al., 2002). The word diversity relating to the language complexity can demonstrate the level of information, the higher the word diversity is, the knowledge is broader (Jensen et al., 2013; Averbeck and Miller, 2014). In communication, people often talk as appropriately as possible to achieve their expectations (Burgoon and Miller, 1985). In terms of business online review, it is important to identify and simulate that particular way of expression to ensure conformity within that group. In general, these studies are conducted on the premise that previous utterance has contextualized the thoughts and expressions of the recipients who are faced with the same scenario; however, they do not touch on the similarities and differences of adjacent recipients that may encounter different types of a product. In this research, we used LET to explain how previous expressions relating to product quality influence the expressions of the recipients.

### Product-Centered Language Style

The content of the online review is able to manifest the review value (Willemsen et al., 2011) because in e-commerce, customers rely on limited knowledge and information they have to make purchase decisions (Steenkamp and Ter Hofstede, 2002). The attributes of the content such as length, rating valence, and affective content directly influence the review expressions (Forman et al., 2008). These review content features also influence the high and low quality of reviews in the eyes of customers (Huang et al., 2015; Liu et al., 2018). Besides, online review content is a source for customers to get various knowledge about products. Through browsing the content generated by previous writers, customers have access to social knowledge about the products they are interested in (Huang and Benyoucef, 2013).

The language style inherent in the linguistic information of online reviews offers cues for customers to assess the product (Willemsen et al., 2011). The signal and observability offered by language style are one of the components to judge the quality. Linguistic variables such as word count, argument quality, review clarity, and readability are sometimes central indicators of product quality (Maslowska et al., 2017). According to Srivastava and Kalro (2019), central linguistic factors primarily consist of “‘manifest’ attributes such as word count, number of sentences, review rating, and number of images, which are easily observable”; “latent” factors are embedded linguistic text features, in particular language style.

Recent research still focuses on ECLS from diverse business-related factors. For example, Yin et al. (2017) found that emotional arousal from a similar language style positively affects the value of the product through the price valence. The direct language contains mental energy, feelings, and motivations and presents intense emotions in post-buyers, considering the relationship of the matching level of function words (Precht, 2000, 2003). Information and emotional process can be used to manipulate by the statement that “the language style of online reviews affects how an audience perceives the reviews, independent of the content of reviews” (Ghahtarani et al., 2020). Based on these studies, some scholars have begun focusing on the relationship between products and online reviews. Product information regarding product quality is more important than emotions, for it reduces customers’ uncertainty (Pavlou and Dimoka, 2006). Useful information is obtained by customers from the non-emotional content, especially the product quality (Jiang and Benbasat, 2007; Liu et al., 2019). Customers usually add their own information to the text model to create a new comment that is in line with the previous reviews (Gernsbacher and Hargreaves, 1988; Zwaan and Radvansky, 1998). In this way, customers develop a relational tie with the cognition of the previous text in terms of the product (Wang and Karimi, 2019). The way of expression reflecting the product quality is one of the indicators that help customers make the judgment of the product quality (Guo et al., 2020). But among these studies, no one explores the features of the language style of the online review in terms of the product itself. In this study, we contributed to the literature that the PCLS is under investigation in the context of Chinese women clothes online reviews.

### Studies Related to Language Style Matching

Language style matching is a technique of text analysis that measures the matching style degree of two or several people in their utterances (Ireland et al., 2011). People who have the same interest or background are likely to make utterances that mirror the sentence structure they have previously heard or read, and the verbal and non-verbal behaviors after a social interaction becomes synchronized (Ireland and Pennebaker, 2010). Such phenomenon is called LSM, which shows the matching level of identical behaviors between one individual and another, either the verbal or the written style (Gonzales et al., 2010).

Language style matching has been extensively used as an approach in various business discourse texts to study the impact and characteristics of consumer comments (Ireland et al., 2011). Using this linguistic method, it is found that figurative language of consumer reviews leads to favorable attitudes to an enjoyable purchase experience (Kronrod and Danziger, 2013); post-reviewers will note down figurative language instead of literal language to show their persuasive influence (Wu et al., 2017); the psychological processes can be identified to examine qualitative features of review text (Liu et al., 2018); mitigated perceived perception/expectancy on certain products is congruent with the negative text acquired by post-reviewers (Burgoon and Poire, 2010; Liu et al., 2018). In recent research, LSM gives rise to the function words used in an online review, by which emotions’ roles are thoroughly studied (Fan and Chen, 2017; Liu et al., 2018; Guo et al., 2020). For example, the sentiment in online consumer reviews negatively or positively affects the degree of synchrony of expressed texts made by recipients (Salehan and Kim, 2016; Yin et al., 2017). The engagement in email references of the librarian’s answers is measured to indicate the holistic assessment of virtual reference services (Agee, 2019). In addition to function words, the impact of topic and focus of interest on post-customers has drawn wide attention with the approach of LSM. Lin and He (2009) proposed a joint sentiment/topic model detecting how simultaneous sentiment is likely to reflect the change of topic theme, the socioeconomic characteristics. The attention focus plays a key role in families, communities, and at work when people happen to talk about a common theme (Chung and Pennebaker, 2007; Ireland et al., 2011). In this study, applying the LSM method in women clothes online reviews in the context of PCLS, we attempted to explore the linguistic characteristics of product-centered texts, that is, what semantic expression makes one group’s reviews different from those of another group.

Language style matching is an algorithm developed mainly to calculate verbal cohesiveness based on an automated textual analysis of words, which can predict the cohesiveness of the language used by various groups (Tausczik and Pennebaker, 2010). Language features including word count, sentence patterns, and verb tense were also related to the groups’ cohesiveness and performance (Gonzales et al., 2010). According to Gonzales et al. (2010), the LSM algorithm is calculated in the following procedures. First, count the absolute value of the proportions of words in a given word category out of the total number of words in each group. Second, divide this value by the sum of each group, where the difference in word usage adjusted for combined word usage in that category between group 1 and group 2 is provided. Then, the result is subtracted by 1 to obtain the similarity.

In this study, we measured PCLS based on the LIWC for product content in the online reviews. Therefore, we employed a refined LSM calculation based on the formula created by Gonzales et al. (2010).

LSM affordable = 1 − [| (G1 − G2) |]/[(G1 + G2 + 0.000001)].

In the above formula, “G1” and “G2” stand for the written texts in the word category of “affordable” by each group of customers. The denominator of 0.00001 is to avoid empty sets in cases of a zero LIWC category’s score. G1 is obtained by calculating the percentages of total content-and-sentiment words in the word category among the total number of words. The result is an 8-dimensional language-style score for each review.

## Methods

### Overview of the Research Method

**FIGURE 1:**
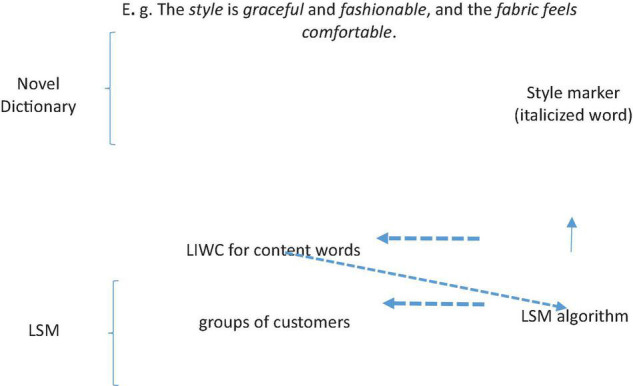
Overview of the research method.

In this study, we proposed an LIWC for content words with the LSM approach in an attempt to demonstrate how the PCLS of a given group of Chinese women clothes online reviews differs from that of the other two groups. The overview of the method is seen in Figure 1. It is composed of two parts. The first part is to build a novel LIWC dictionary for content words, ready for the calculation of LSM. The second part is to calculate the language style of three groups of Chinese women clothes customers based on the LSM algorithm, and the results are used to compare general features of PCLS, that is, whether convergence and divergence of language style exist in the online review in terms of age group. To be specific, there are four procedures: (1) new content words representing the linguistic content words (product-centered words) are established to adapt to the online women clothes reviews; (2) the product-centered words are based on words that reflect the product categories most talked about in women online reviews; (3) the LSMs of three groups of women customer reviews are calculated; (4) the results of LSMs are compared with each other to show the level of cohesiveness and likelihood. We gave special consideration to the PCLS in terms of LSMs because customers believe that product quality can bring them a more useful assessment of the product than the sentiment does (Pavlou and Dimoka, 2006).

### Reviews of Chinese Women Clothes

To make the samples as wide as possible with regards to Chinese women online reviews, we established a corpus of 32,667 online reviews written by women clothes buyers, from Taobao, an online e-commerce platform of Alibaba in China, spanning from 2018 to 2020. Information relating to product quality includes brand, manufacturer, size, color, texture, etc. More importantly, the online reviews vary with a host of factors such as age, community, and personal hobbies. Among these factors, age is one of the most influencing factors in women clothes purchases (Mirowsky and Ross, 2001). Our data are extracted based on the age group as set out by Taobao. The Taobao division of the age group is set by its own system of division, which is not in line with the division standards of the prevailing standards, for example, the standards of the World Health Organization (2010). Therefore, we followed the typical Taobao division of demography by age and classify them into three age groups: 18–25, 25–45, and 45–65, respectively, representing young, middle, and seniors. In each age group, the online reviews are expressed under considerable and enormous brands of women clothes. To address the impact of brands on online reviews, we selected those brands’ reviews by sales volume not lower than the top 10 brands, which are listed on Taobao’s web page. Then, the reviews are randomly taken according to brands as shown in Table 1.

**TABLE 1:** Data collection source.

Group No.	No. of reviews	Age span	Top brand(s) by volume	Age group based on Taobao division
G1	10,553	18–25	Peacebird, Tyakasha, Handu Group, La Chapelle, etc.	Young
G2	11,096	25–45	Ochirly, Prich, Hopeshow, Uniqlo, etc.	Middle
G3	11,018	45–65	Cadence, Ms. Tang, Cabenzioe, Sonmellny, etc.	Seniors

### Content-Word Linguistic Inquiry and Word Count

According to Pennebaker (2015), the LIWC is one of the core parts of LSM. LIWC is used to count and classify words. With the LIWC program, each word can be encoded and compared to see if it belongs to one of the words classified under a certain category in the dictionary. For example, “linen” is coded as a content word of material category. Then, “linen” is compared to see if “linen” is in the dictionary. LIWC produces the percentage of each LIWC category and usage frequency. The updated LIWC program (Pennebaker, 2015) is a 90-plus category dictionary composed of almost 6,400 words, word stems, and emotions which additionally include one or more word categories or sub-dictionaries. Most prominent in the dictionary is “21 standard linguistic dimensions (e.g., percentage of words in the text that are pronouns, articles, and auxiliary verbs) and 41-word categories tapping psychological constructs (e.g., affect, cognition, biological processes, and drives)” (Pennebaker, 2015, p. 4).

The amazing part of LIWC is that it expands into aspects of words and gets updated. Psychology and children’s language LIWC (Hartley et al., 2003) have been included. Recently, the causal words and insight words under the category of cognition are included as well to show the level of appraisal (Kross and Ayduk, 2008). As for the research on an online review, current LSM is based on the emotional aspect, in particular the function words of the emotional aspect in the LIWC (Leończyk, and Aleksandra., 2016; Fan and Chen, 2017). Although some scholars have argued the social influence on consumers from the perspective of economic status, gender, etc., the reliability of language style is dependent on the data from emotional functional words (Willemsen et al., 2011; Cheng and Ho, 2015). In brief, there is no LIWC categorized by words in regard to product quality at present.

According to Tausczik and Pennebaker (2010), content words and function words play an equal role in the evaluation of language style. Style or function words are those words such as pronouns, prepositions, articles, conjunctions, and auxiliary verbs. Content words include nouns, regular verbs, adjectives, and adverbs, which convey the content of communication (Haspelmath, 2001). In this study, based on the features of our data and business practice, we developed a word dictionary of content words (Table 2) by a combination of relevant studies (Yu, 2012; Zhang, 2019). Our dictionary is an 8-category product content dictionary, which is affordability, brand, color, clothes function, materials, size, garment style, and workmanship, respectively. For example, words such as “slim,” “warm,” and “young” are grouped into the category of clothes function while words such as “linen,” “silk,” and “texture” are classified into the material category.

**TABLE 2:** Product content category used to calculate language style matching (LSM).

Dictionary category	Examples
Affordable	Cheap, worth, worthwhile
Brand	Clothes, garment
Color	Red, yellow
Clothes function	Slim, beautiful
Material	Texture, linen, silk
Size	Big, size
Clothes style	Fashion, style
Workmanship	Quality, workmanship

## Results

### General Results

Based on our corpus of 32,667 online reviews in terms of three age groups, we acquired Table 3, which shows the LIWC score, LSM scores, and LSM mean.

**TABLE 3:** Sample LSM for online review of female garments.

LIWC category	LIWC score			LSM scores			Mean LSM
	G1	G2	G3	G1/G2	G1/G3	G2/G3	
Affordable	0.00	1.82	1.39	0.00	0.00	0.87	0.29
Brand	0.74	1.22	2.79	0.75	0.42	0.61	0.59
Color	2.57	1.22	1.05	0.64	0.58	0.92	0.71
Functional	5.51	10.03	9.41	0.71	0.74	0.97	0.81
Material	0.37	2.43	6.97	0.26	0.10	0.52	0.29
Size	0.37	1.22	5.57	0.46	0.12	0.36	0.32
Style	4.41	6.99	3.14	0.77	0.83	0.62	0.74
Workmanship	0.74	3.34	1.74	0.36	0.59	0.69	0.55

**FIGURE 2:**
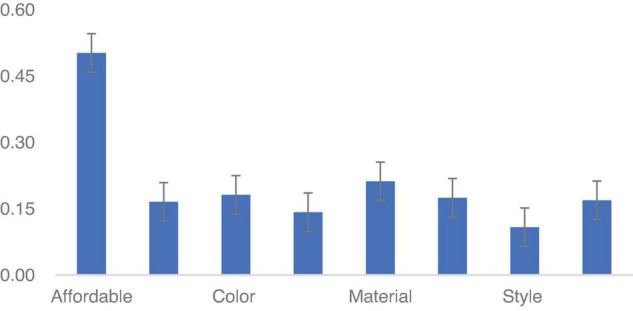
The SD of three language style matching (LSM).

To test the validity of these data, we conducted a standard deviation (SD) for 8-category words of three groups. The SD ranges from 0.11 to 0.5, which means that the data collected are acceptable (refer to Figure 2).

### The Diverse and Polarized Product-Centered Language Style

The disparity of each group in terms of the specific categories of the product indicates that the PCLS of women clothes buyers is diverse in general. From Table 3, the LWIC within the young women (G1) shows three high figures, namely, function, style, and color, at 5.51, 4.41, and 2.57, respectively, while other aspects stay at a stunning figure lower than 1, with price at 0. This overwhelming way of expression with regards to a product indicates that young women clothes buyers, though cognitively influenced by previous customers, are likely to expressively comment on the function and style of a product than on price. Likewise, we can get the linguistic features of middle-aged women (G2) and senior women (G3). The PCLS of middle-aged women is evenly distributed with three high figures, namely, function (10.03), style (6.99), and workmanship (3.34), which manifests that middle-aged women have a more equilibrium view on clothes and tend to pose a higher demand for quality (workmanship) than young women (G1). As for G3, the PCLS is more evenly distributed with four high figures, that is, function (9.41), material (6.97), size (5.57), and style (3.14), which shows that senior women buyers hold a comprehensive look at clothes with a special concern for the size and style.

**FIGURE 3:**
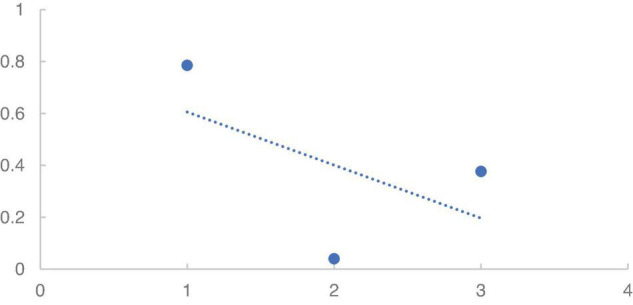
The linear regression of correlation of three language style matching (LSM).

To further show the general trend of PCLS, we conducted the linear regression as shown in Figure 3. Figure 3 shows the linear regression of the correlation of three LSMs. The *X*-axis stands for the three age groups, while *Y*-axis means the correlation coefficient. It is seen that the PCLS presents a downward trend in terms of three groups of women online buyers, which means that the PCLS diverges or drops in matching in terms of age groups.

### The Priority of Product-Centered Language Style

In this study, we posited that the arrangement of product categories is an indicator of the language style a customer employs to demonstrate his or her identity, and in other words, the priority of texts/words relating to product categories affects the way of expression.

To acquire the PCLS of women clothes buyers in terms of priority and importance, we applied the results of LSM to the statistical analysis function of percent rank and get the importance list of the eight categories of women clothes reviews (Table 4).

**TABLE 4:** Product-centered language style (PCLS) priority rankings between groups.

Rank	Significance (%)	Category	G1/G2 LSM	Category	G1/G3 LSM	Category	G2/G3 LSM
1	100.00	Clothes style	0.77	Clothes style	0.83	Clothes function	0.97
2	85.70	Brand	0.75	Clothes function	0.74	Color	0.92
3	71.40	Clothes function	0.71	Workmanship	0.59	Affordable	0.87
4	57.10	Color	0.64	Color	0.58	Workmanship	0.69
5	42.80	Size	0.46	Brand	0.42	Clothes style	0.62
6	28.50	Workmanship	0.36	Size	0.12	Brand	0.61
7	14.20	Material	0.26	Material	0.1	Material	0.52
8	0.00	Affordable	0	Affordable	0	Size	0.36

Table 4 shows that the overall importance of the eight categories is rated based on the results of LSMs, with a breakdown of percentage from 100 to 0%. One way to interpret Table 4 is that by the same percentage of importance, three groups show a different priority/sequence of product categories. For example, the priority of G1/G2 is clothes style, brand, clothes function, color, size, workmanship, material, and affordability, which are different from that of G1/G3, that is, clothes style, clothes function, workmanship, color, brand, size, material, and affordability. If the significance is set as 50% manually to show the high (>50%) and low attention focus (<50%), we can see that color and function are the high “categories” shared by the three groups. The second way to interpret Table 4 is to look up the percentage of a certain product category, for example, “material.” Fortunately, the category of “material” is the only one that marks the same ratio at 14.2%, signifying the “only” shared interest of focus among the three groups. As the percentage of product categories is indicative of the focus of interest from customers, we can see that the different percentage by significance shows a clear-cut priority with regards to the way of expression of women clothes reviewers.

## Discussion

The first question of this study is to describe the key language features of women clothes online reviews when they comment on issues relating to a product. As shown in the previous section, diverse and polarized are the key features. We attributed the diversity of PCLS to the phenomenon called the insider language style of a speech community. Fairclough, 2003 defined insider languages as the marked language that serves to construe and reinforce the identity of a speech community. In online reviews, customers usually add their own information to the text model, which actually creates a new comment in line with the previous reviews (Gernsbacher and Hargreaves, 1988; Zwaan and Radvansky, 1998). Insider customers are sensitive to the language style of their community and follow the text rules that demonstrate such factors as the density of words and the text of social cognition (Cheng and Ho, 2015) or social variables such as gender, religion, and social position (Deckert and Vickers, 2011). To be specific, the young women clothes buyers (G1) identify themselves as the group of people who cares least about money, which is reinforced within the community of G1 and eventually forms a special language style.

In addition to diversity, we found that women reviews show a polarized trend by division of the group. We calculated the average mean of three LSM (0.49, 0.42, and 0.70) and then apply the correlation function. The results are 0.78, 0.04, and 0.37. By the definition of the correlation function, a higher correlation value means higher relatedness of the two variables. The G1/G3 correlation (0.04) shows that G1 and G3 have the least matching in language style, which can well explain the group disparity between young and seniors, whereas the G1/G2 correlation (0.78) shows relatively high synchrony in language style, which represents the smooth transition from young- to middle-aged woman. This result is of significance to clothes manufacturers in that products designed for young women can be branched out to middle-aged women in business promotion strategy, which may greatly increase the demography coverage of target customers.

This polarized feature can also be explained by the high and low language style matching in women clothes reviews with regards to product quality. According to Tausczik and Pennebaker (2010), the LSM score is on a scale of 0–1.0. A score of zero indicates no matching in the language style while 1.0 means high cohesiveness. Scores of 0.60 or below are usually considered low matching, and scores of 0.85 or above are deemed as signs of high matching. High and low matching levels have been proved valid in different fields. For example, high-level matching exists among customers of different nations who speak different languages when they comment on the product (Schuckert et al., 2015). Librarians can linguistically generate high-level matching feedback to the comments of the audience (Agee, 2019). The desire to enhance or mitigate the impact of language style may influence recipients who eventually produce a high or low synchronized way of expression (Giles et al., 2007). Hilte et al. (2020) proved that a certain degree of high LSM exists in two genders in teenagers’ social media writing. Table 3 shows that the only category of high matching level (>0.85) appears in G2/G3, which are clothes function, color, and price (0.97, 0.92, 0.87) while there is none in G1/G2 LSM and G1/G3 LSM. This points out that G2/G3 online reviews influence each other higher and stronger in the categories (garment function, color, and price) than other product categories, which, in the eyes of a company, may locate the “shared” aspects of a product among different customer groups.

We assumed that the declining trend of language style matching degree in Figure 3 is largely attributed to the weakened expectancy of information or the stronger role of insider language. As language expectancy theory puts it, if people sense the style of community speech and get affected, they may translate the language style of previous comments into their own language style (Burgoon and Miller, 1985). But this LET is conditioned by the *ad hoc* fact that customers are able to identify the language style. In case of failure to the identification of prior comments, the chances of encoding are slim. Since G1 is unlikely to identify the encoded language style of G3, which may cause the low initiation of language expectancy by a host of factors such as leadership, honesty, status, age, and culture (Chung and Pennebaker, 2007), G1 may not mimic the text model of G3 and eventually comment very differently.

For the second question on the makeup of PCLS, we referred to the concept of word of significance. Groves et al. (2000) stated that the way that a potential participant responds to a product depends on the importance placed on an element of the product. It is inferred that if the importance is placed on a host of linguistic elements with different degrees of priority, and such importance is identified by the post-customer and rearranged into his online review with extra information, the importance placed on a host of elements is, in fact, the linguistic salience of prioritized product features by importance. Since people who think more about product quality will repeat the elements of product quality and be controlled in their text expression (Tausczik and Pennebaker, 2010), customers can identify and track what information they are interested in which is linked to product quality.

The fact that customers use priority PCLS to gain useful information is related to psychological linguistics. The depth of thinking or attention is reflected in the language style, which links thoughts (Tausczik and Pennebaker, 2010). In other words, the language style is reflected in thinking that correlates to how frequently a word or a group of words are mentioned. In essence, language style is closely related to attention focus, which is one of the key factors that contribute to the construction of language style (Pennebaker, 2011). The self-reference of pronouns “I” in the political advertisement is a representation of the idea of being independent (Gunsch et al., 2000). The use of “we” is linked with “better” marriage (Simmons et al., 2005). The style differences of the verb tense are the result of the speaker’s attitude toward reality (Pasupathi, 2007). These studies attach importance to using tokenized words to differ the language style, but none focuses on the arrangement of the tokenized words, let alone the PCLS priority.

It is interesting to see from Table 4 that the ranking/sequence of G2/G3 against that of G1/G2 is 2 against 4, which represents the ratio of the language matching degree of middle-aged women/senior women vs. that of young women/middle-aged women. The higher priority of “color” in terms of middle-aged women/senior women implies that people of older ages like to dress in brighter and glossier clothes. We reasoned this to be the combination of individual focus and situational focus. The language style of a certain group is featured with the two characteristics as follows: the uniqueness of the language style of a person and the shadowed influence from other groups (Krapp, 2002; Hidi and Boscolo, 2006). Individual focus results from an individual’s enthusiasm and tendency toward specific information and objects. Situational focus is activated by the surrounding environment such as the emotions of others. In the case of women of older ages, they are influenced by the contextual situation of the middle aged group (G2) in the choice of “color.” Also, the fact that senior women mention more frequently the word “color” is attributed to the individual personality because the self-focus inherent in language use that “differs with age, sex, personality, and mental health” (Tausczik and Pennebaker, 2010. p. 36) can be identified from their linguistic characteristics.

## Conclusion

Recently, scholars studied the language style used in product online reviews (Huang et al., 2015; Liu et al., 2018), solely based on the function words, which demonstrates that the emotional language style embedded in function words is related to the product. However, a large part of online reviews is concerned about product quality instead of emotions. In this study, we built a corpus of Chinese women clothes online reviews to explore the PCLS. A novel LIWC dictionary covering eight categories of product quality is sorted and established, which, in the practical sense, adds a new dimension to the existing LIWC dictionary.

With the product-category LIWC and the approach of LSM, we are able to find that the PCLS of women clothes buyers lies in (1) the diverse but polarized way of expression among different groups of buyers. The correlation function of all LSMs shows that young and seniors have the least matching in language style while middle age and seniors show a high similarity in PCLS; (2) despite the polarized language style, there exists a priority of language style relating to a certain product category, and such priority of PCLS is influenced by how customers weigh product categories in their heart. In this regard, PCLS provides a new dimension to study the language style of online reviews in the context of the product.

As pointed out, the language style of online review is to boost more effective communication. In this regard, the language style of online review may be more effective to convey customer expressions if it is used with the body signal (Poggi et al., 2013) and facial expressions (Heisler et al., 2003). This stance on effective signals may be taken into account for the future study regarding the PCLS.

This research contains practical benefits not only for women clothes industry but also for consumer management dealing with online reviews. Consumers, regardless of men or women, use language style in online consumer reviews to classify reviews and show their identity (Schindler and Bickart, 2012). The PCLS of women clothes online reviews, featuring diversity and polarity among different groups, can help companies quickly find the polarized PCLS and the transition of PCLS, which can be an aid to the product promotion strategy and product line design. Moreover, the priority PCLS offers a platform for companies to indiscriminately look into the importance of the product category from the linguistic perspective, where feedback (online reviews) of each product category can be quantitatively weighed by the same significance. In general, this quantitative PCLS can help companies identify those crucial product categories and improve product design and promotion strategy.

## Data Availability Statement

The raw data supporting the conclusions of this article will be made available by the authors, without undue reservation.

## Author Contributions

XW and CM are the principal authors of this article, contributed equally to the conception of the article and to the review and critical analysis of relevant scientific literature, wrote a first version of the manuscript, arranged new sections, and produced a final version of the manuscript incorporating changes in response to the helpful suggestions of the reviewers. HL contributed to the conception, to the review and critical analysis of scientific literature on the subject, collecting data and building the corpus and she edited the figures, tables, and references in each version of manuscript. AN contributed to review the different versions of the manuscript and to polish the language. QW applied the mathematical method of percent rank function and interpreted from the perspective of social economics. All authors contributed to the article and approved the submitted version.

## Conflict of Interest

QW was employed by ByteDance Ltd. The remaining authors declare that the research was conducted in the absence of any commercial or financial relationships that could be construed as a potential conflict of interest.

## Publisher’s Note

All claims expressed in this article are solely those of the authors and do not necessarily represent those of their affiliated organizations, or those of the publisher, the editors and the reviewers. Any product that may be evaluated in this article, or claim that may be made by its manufacturer, is not guaranteed or endorsed by the publisher.
